# Associations Between Internet Addiction and Gender, Anxiety, Coping Styles and Acceptance in University Freshmen in South China

**DOI:** 10.3389/fpsyt.2021.558080

**Published:** 2021-05-31

**Authors:** Xiaoxiao Shan, Yangpan Ou, Yudan Ding, Haohao Yan, Jindong Chen, Jingping Zhao, Wenbin Guo

**Affiliations:** ^1^Department of Psychiatry, National Clinical Research Center for Mental Disorders, The Second Xiangya Hospital of Central South University, Changsha, China; ^2^Department of Psychiatry, The Third People's Hospital of Foshan, Foshan, China

**Keywords:** internet addiction, students, prevention, gender differences, anxiety, coping style, acceptance

## Abstract

**Objective:** Internet addiction (IA) has become a global public health issue. Although previous studies revealed several risk factors related to IA, most of them focused on the western societies. The present study assesses the relationships between gender and other factors with IA in university freshmen in the South China.

**Methods:** A total of 3,380 first-year college students (1,995 males and 1,385 females) participated in an evaluation of their experiences surfing on the Internet. We investigated the severity of IA in the participants by considering their psychological characteristics, such as acceptance, anxiety levels, and coping styles. Then, we compared the results between males and females and between those in addiction group (Chinese Internet Addiction Scale, CIAS, scores≥64) and non-addiction group (CIAS scores ≤27). We also conducted a logistic regression analysis to detect the relationships between severity of IA and psychological characteristics and gender differences.

**Results:** We observed that males showed significantly higher scores in CIAS than females. The addiction group exhibited significantly higher state anxiety and trait anxiety, and experienced less acceptance of self and others and acceptance by others, and adopted less positive coping style and preferred negative coping style than non-addiction group. The logistic regression analysis revealed that three factors (negative coping styles, acceptance of self and others, state anxiety levels) had a significant association with more severe IA.

**Conclusion:** Gender differences affect the severity of IA in the first-year students in South China. Males with state anxiety and negative coping styles deserve attention because they are likely to be addicted to the Internet. Thus, health practitioners should perform efficient strategies while considering gender differences to precaution first-year college students with the risk factors for IA.

## Introduction

The number of Internet users increases rapidly worldwide given that the Internet altered people's lifestyles through its advantages in communication, socialization, and even online education (1). However, Internet addiction (IA), as a fast-growing addictive behavior, has become a significantly global health problem (2). The concept of IA, also termed as pathological Internet use, Internet overuse, and problematic Internet use by various researchers, is an interpretation for uncontrollable, disruptive use of the Internet with psychological dependence. People manifest this behavior as significant/overuse, withdrawal, tolerance, negative influence/conflict, desire, and emotional regulation (3, 4). It may cause difficulties in maintaining real-life relationships, impair daily activities, develop poor dietary habits, worsen school performance, and interfere with professional functioning (5, 6). And IA could increase the risk of psychological distress, such as depression, anxiety, low self-esteem (7–9). Others showed its relations with behavior problems, such as aggression, self-hurt behaviors, and even suicide (7, 10).

Education institutions have efficiently used Internet instruments to train students, especially in academic lectures, interaction with teachers, classroom discussion, homework, and sharing of learning resources (11). However, a recent meta-analysis showed that the IA overall prevalence rate in Chinese college students was 11%, which was higher than the other countries, such as Japan (3.7%) and Italy (4.3%). Male students (16%) had a higher rate of IA than their female counterparts (8%) in China (12). Chen et al. showed that the prevalence of IA in grades 3, 5, and 8 students from primary to middle school (aged < 18) was above 10% in Taiwan (13). The prevalence rate for IA in adolescents in Japan was ranging from 2.8 to 9.9% (14). Longitudinal studies have revealed that the incidence rate for junior high school students was 2.5–3.6% after a 2-year follow up in China (15). A cross-cultural epidemiological study showed that Asian adolescents have significantly higher rates of IA than their counterparts in the Western countries (16).

Regarding the gender influence on IA, previous studies produced inconsistent results (17–19). Alamgir et al. found that IA has no relation to gender but has a negative association with physical activity (19). Marzilli et al. concluded similar results (20). However, a recent meta-analysis showed that male Chinese college students (16%) have a higher rate of IA than their female counterparts (8%) (12). Shek et al. revealed that gender difference remains a factor for high Internet addictive behavior among high school teenagers in Hong Kong (21). By contrast, Chiu et al. found that female college students in Taiwan are more likely to develop IA through their smartphones than male students (22). A variety of factors, such as personal habits, access to the Internet, cultural values, and institutional policies, may have an attribute to different results regarding gender and IA. Moreover, the incidence of Internet related mental health problems is significantly higher in only children than in those with siblings. IA has more effect on families with only children and single-parent families (23). Accordingly, studies should consider family factors when evaluating IA.

Anxiety is a dangerous element for IA (24, 25) and is one of the personality characteristics of behavioral addiction tendency (26). Cross-sectional studies have shown that IA has a significant relation to anxiety (7, 10, 27). A Meta-analysis also revealed similar results (25). Both symptoms of pathological Internet use and emotional troubles could lead to a vicious cycle that favors the continuation of pathological Internet use (28). In addition, IA studies showed its relations to negative coping styles. Mcnicol et al. found that avoidance coping style has a positive correlation with IA (29). Using less effective stress coping styles might increase the risk of IA after 1 year follow up in college students (30). People who commonly use less effective stress coping strategies might overuse the Internet in order to get rid of or vent stressful events (29, 30). Although previous studies reported the risk factors related to IA, the understanding to it may still be incomplete. Whether the risk factors related to IA are culture-specific remains unclear, as most researches on risk factors have focused on the Western societies. The coping styles, acceptance of self and others, and the relationship between teenagers and their classmates or teachers have a great effect on the psychological status of individuals, which may affect their Internet use. From the perspective of China's development index, the development level of different regions varies greatly, and the Internet resource distribution is uneven. To the best of our knowledge, no study has explored the combined effects of emotional state, coping styles, and acceptance of self and others as leading to IA in first-year college students in the South China. Hence, we think the data in the present study may provide a significant contribution to this field.

Young adult Internet users, particularly college students, are more likely to be addicted to the Internet than older Internet users (31). Psychological and environmental factors in college students' lives might make them easily vulnerable to IA (32). Several reasons might attribute to the situation: (1) universities offer free and unlimited Internet access; (2) college students have considerable free time; (3) college students are out of their parents' control for the first time, with no one monitoring or censoring what they say or do online; (4) young college students have new problems adjusting to college life and making new friends, often seeking friendship through different applications in the Internet; (5) college students are training to use various technological inventions and applications, particularly the Internet (33). This study aims to explore the relationship between IA and gender, coping styles, anxiety, and acceptance in the first-year college students. We expected that these factors could predict the consequences of IA in first-year college students in the South China. We hypothesize that gender differences exist in IA, wherein males may have a higher risk of IA than females. We also hypothesized that higher levels of anxiety, negative coping styles, and less acceptance of self and others might predict a higher risk of IA.

## Methods

### Participants

We conducted a cross-sectional study in Changsha, one of the largest cities in the South China, from October 10 to December 20, 2019. Inclusion criteria were as follows: (1) active Internet users for more than 1 year; (2) college freshmen agreed to complete the questionnaire. The exclusion criteria were those who were unwilling to complete the questionnaires or had no access to the Internet. Two master level research assistants spearheaded the recruitment of the participants. They carefully explained the research purposes and procedures and highlighted confidentiality problems to obtain a written informed consent from the participants. A total of 1,995 males and 1,385 females participated in this study. The participants were 16–24 years old. The average age of the participants was 19.8 years old. Then, they were divided to two groups, including the addiction group and non-addiction groups according to the Chinese Internet Addiction Scale (CIAS). Concerning the procedural side, the researchers first explained to the students the purposes of the research. Then, before handing out the questionnaires to the willing participants, the research assistants asked for their informed consent (An additional informed consent was obtained from the parents or guardians for those aged below 18 years old). During the survey, the research assistants instructed the participants to sit separately, stay quiet, and not participate in discussions.

Our study involving human participants obtained approval from the Ethics Committee of the Second Xiangya Hospital of Central South University and the university's institutional review board and executed in line with the Helsinki Declaration.

### Measurements

#### CIAS Measure

CIAS was used to evaluate the severity of IA in the participants. CIAS is one of the most widely applied assessments on IA with a good internal consistency (16). CIAS is a validated evaluation for the main symptoms of IA and physical and social problems with the Internet (34), which has been widely applied as a criterion for IA in Taiwan and mainland China (35–37). This scale includes 26 items on a four-point Likert scale that assesses five dimensions of Internet-related problems, involving compulsive use, tolerance, withdrawal, interpersonal relationships, and time management and health, with a total score ranging from 26 to 104 (34). The reliability and validity of this scale were good (Cronbach's Alpha, 0.79–0.93) (34). The higher the CIAS score, the higher the severity of IA. The cut-off point of screening in this scale, with total scores ranging from 26 to 104, was 57/58 (38). Those with a score of 63/64 or more are considered Internet addicts (39, 40). The Cronbach alpha of this scale in the present study was 0.935.

#### State-Trait Anxiety Inventory

STAI was used to evaluate the severity of anxiety. It is a 40-item questionnaire on a four-point Likert scale, which comprises two separate subscales that measure the participants' state and trait anxiety levels, respectively, with good reliability and validity (α = 0.87 for state anxiety, and α = 0.85 for trait anxiety) (41, 42). Higher scores indicate increased state or trait anxiety levels. The subscale consists of 20 items, with scores ranging from 20 to 80. The “state anxiety” used in the present study shows how the participants feel “at the moment,” whereas “trait anxiety” shows their “general feelings.” The Cronbach alpha of this scale in the present study was 0.91.

#### Acceptance of Others

AO was compiled by American psychologist Fey (43) and translated and revised by a domestic scholar Xiaodong Fan. This scale, including 20 items on a five-point Likert scale with total scores from 20 to 100, assesses the participants' acceptance of self and others and acceptance by others (43, 44). The higher the two subscale scores, the higher the level of the participants' acceptance of self and others and acceptance by others. The split-half reliabilities for acceptance of self and others and acceptance by others were 0.90 and 0.89, respectively (43). The Cronbach's alpha coefficients for acceptance of self and others and acceptance by others in present study were 0.703 and 0.603, respectively. The split-half reliability in the present study was 0.758.

#### Simplified Coping Style Questionnaire

SCSQ, including 20 items on a four-point Likert scale with scores on each item ranging from 0 to 3, assesses the participants' positive and negative coping styles (45, 46). The higher the two subscale scores, the higher the level of the participants' positive or negative coping styles. The positive coping subscale includes items 1–12, which reflects the features of individuals' positive coping style in face of pressure. The negative coping subscale includes items 13–20, which reflects the features of individual negative coping style (47). The Cronbach's alpha coefficients for positive and negative coping styles were 0.89 and 0.78, respectively (45). The Cronbach's alpha coefficients for positive and negative coping styles in the present study were 0.637 and 0.626, respectively. The Cronbach's alpha of this scale in the present study was 0.61.

### Statistical Analyses

All participants completed the four inventories after receiving instructions from the researchers. We used chi-square to analyze the difference in gender and family factors, including single-child family, adoptive parent condition and parents being alive between male and female groups. Then, we compared gender differences in age, CIAS, STAI, AO, and SCSQ scores by two-sample *t*-tests. Previous studies showed the cut-off point of 63/64 or more in CIAS has good accuracy, sensitivity and specificity for diagnosing IA (39, 40). To study the relationship between IA and other factors, including anxiety, acceptance, and coping styles, the subjects were divided into two groups, including the addiction group (CIAS scores ≥ 64) and the non-addiction group (CIAS scores ≤ 27). The matching non-addiction participants were selected from the lowest scores of CIAS. We also compared the differences in the STAI, AO, SCSQ, and CIAS scores between addiction group and non-addiction group by two-sample *t*-tests.

We analyzed the correlations between the severity of IA and STAI, AO, and SCSQ scores in male and female groups by using partial correlation analysis while correcting the confounding factor of age.

We further used a logistic regression to evaluate the relationship of IA in the predictive model between gender differences and other variables, including STAI, AO, and SCSQ, while controlling the effects of age. A *P*-value of <0.05 was statistically significant.

## Results

### Demographic and Clinical Psychological Characteristics

A total of 1,995 males and 1,385 females participated in this study. Among them, 33 lacked a record of their age who were not removed from the study, and 98 individuals lacked a record of whether they were a single child. Moreover, 16 participants lacked a record of whether they had stepparents; 109 participants lacked a record of whether their father is alive; 93 participants lacked a record of whether their mother is alive; 17 participants lacked a record of their satisfaction degree for the admission. The mean age of male and female participants was 19.91 ± 0.86 and 19.73 ±0.73, respectively (*P* < 0.05). For the family factor, only a small percentage of participants lost their father or mother, and no significant differences were found in the parents' alive condition between the male and female groups. Significant differences were evident in single-child families between the male and female groups (*P* <0.05), more participants in the male group were not single-child (male group: 1,141 for not single-child; and 785 for single-child). By contrast, no substantial differences were evident in the adoptive parent condition between the two groups. Most participants had no adoptive parents (male: 2.67/97.33%; and female: 3.56/97.44%). The details were offered in Supplementary Table 1. The following reasons may account for the unrecorded familial data: first, some participants might fail to complete the familial data; second, some participants were unwilling to finish them because family data were some personal privacy. The satisfaction degree of admission is used to measure the degree that the college freshmen were satisfactory with the admission of the current college. The survey of satisfaction is not only a rational evaluation of the students' perceptions and expectations, but also the psychological representation of college students' learning and attitudes, feelings and experiences. The satisfaction degree of admission may influence the emotion and behavior of college students. And it plays an important role in improving the overall quality of education and promoting education reform. For the satisfaction degree of the admission, 724 participants were very satisfactory for the admission; 1,462 participants were fairly satisfactory; 640 participants felt general; 404 participants were not quite satisfactory; 49 participants did not care for it; and 84 participants were very unsatisfactory.

### Psychological Characteristics of College Freshmen by Gender

Males showed significantly higher scores in CIAS than females (*P* < 0.05), which indicated that the former had a higher risk factor of IA than the latter. Females experienced more acceptance of self and others (*P* < 0.05) and acceptance by others (*P* = 0.003) and adopted more negative coping styles (*P* = 0.004) than males. No gender differences were evident in state and trait anxiety levels and positive coping styles (Ps > 0.05). The details were offered in Supplementary Table 2. We have also conducted the comparison of scale variables between male and female groups using Wilcoxon tests of two independent samples. The results were offered in Supplementary Table 3.

### Correlation Analysis

**Figure 1:**
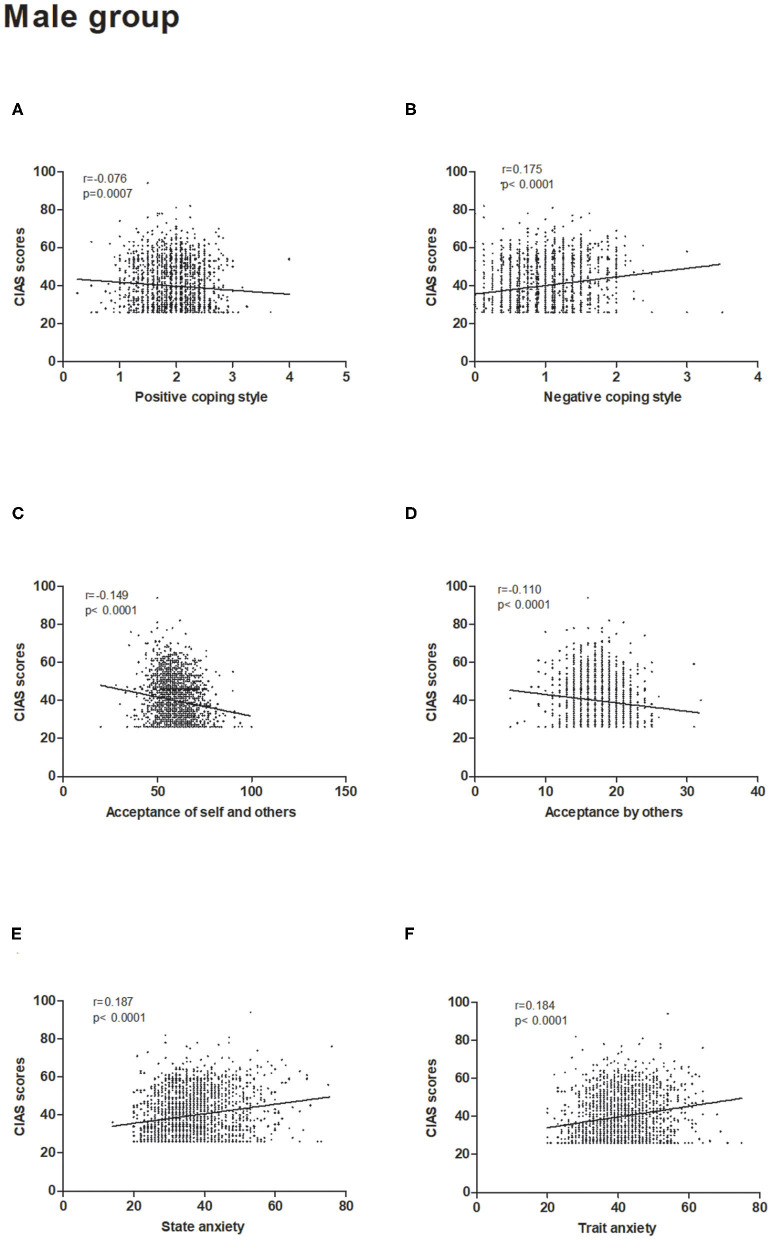
Correlations between the severity of Internet addiction and psychological characteristics in the male group. **(A,C,D)** Negative correlations were observed between CIAS scores and acceptance of self and others, acceptance by others and positive coping style. **(B,E,F)** Positive correlations were observed between the CIAS scores and negative coping style, state anxiety and trait anxiety. CIAS, Chinese Internet Addiction Scale.

**Figure 2:**
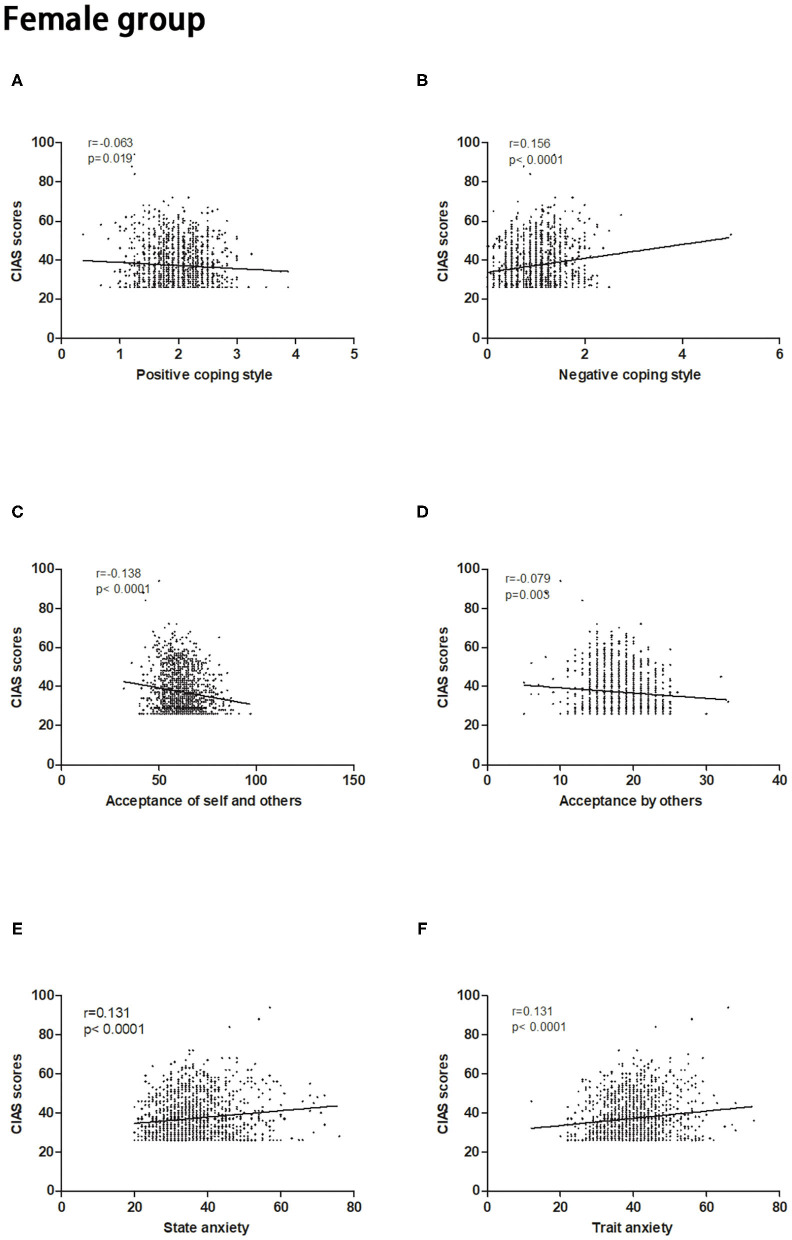
Correlations between the severity of Internet addiction and psychological characteristics in the female group. **(A,C,D)** Negative correlations were observed between CIAS scores and acceptance of self and others, acceptance by others and positive coping style. **(B,E,F)** Positive correlations were observed between the CIAS scores and negative coping style, state anxiety and trait anxiety. CIAS, Chinese Internet Addiction Scale.

The CIAS scores in the male and female groups had significant correlations with acceptance of self and others (male: *P* < 0.05; female: *P* < 0.05), acceptance by others (male: *P* < 0.05; female: *P* = 0.003), positive coping styles (male: *P* = 0.0007; female: *P* = 0.019), negative coping styles (male: *P* < 0.05; female: *P* < 0.05), and state and trait anxiety levels (male: *P* < 0.05; female: *P* < 0.05) after controlling the confounding effects of age (Figures 1, 2). The *p*-values were significant while correlation coefficients were very weak (*r* < 0.20) because of the high number of participants in our study. The details of correlation analysis result were offered in the Table 1. In addition, simple correlation coefficients among all variables in the study have been calculated and the results were offered in the Supplementary Table 4.

**Table 1:** The correlations between CIAS scores and related factors in the male and female groups.

	**Group**	***R***	***P***
Acceptance of self and others	Male	−0.149	<0.0001
	Female	−0.138	<0.0001
Acceptance by others	Male	−0.110	<0.0001
	Female	−0.079	0.003
Positive coping styles	Male	−0.076	0.0007
	Female	−0.063	0.019
Negative coping styles	Male	0.175	<0.0001
	Female	0.156	<0.0001
State anxiety levels	Male	0.187	<0.0001
	Female	0.131	<0.0001
Trait anxiety levels	Male	0.184	<0.0001
	Female	0.131	<0.0001

*CIAS, Chinese Internet Addiction Scale*.

### Comparisons Between Addiction Group and Non-addiction Group

We divided the participants into two groups, including addiction group (CIAS scores ≥ 64, about 477 persons) and non-addiction group (CIAS score ≤ 27, about 572 persons) based on the cut-off point of 64 for diagnosing IA (39, 40). The addiction group exhibited significantly higher state anxiety and trait anxiety (*P* < 0.05), and experienced less acceptance of self and others and acceptance by others (*P* < 0.05), and adopted less positive coping style (*P* < 0.05) and preferred negative coping style (*P* < 0.05) than non-addiction group, indicating that IA was associated with emotional and behavior disorders (Table 2).

**Table 2:** Comparisons between addiction group and non-addiction group.

**Measure**	**Addiction group (*n* = 477)**	**Non-addiction (*n* = 572)**	***p***
State anxiety	39.61 ± 9.11	35.29 ± 8.43	<0.001
Trait anxiety	42.72 ± 7.76	39.42 ± 7.89	<0.001
Acceptance of self and others	57.82 ± 7.40	61.21 ± 9.33	<0.001
Acceptance by others	16.93 ± 2.78	17.70 ± 3.15	<0.001
Positive coping style	1.89 ± 0.44	1.99 ± 0.44	<0.001
Negative coping style	1.10 ± 0.52	0.87 ± 0.47	<0.001
Gender (male/female)	70.44/29.56% (336/141)	66.96/33.04% (383/189)	0.227

*P-values were obtained by two-sample t-tests except for gender (a Chi-square test)*.

*CIAS, Chinese Internet Addiction Scale*.

### Factors Related to IA

We used a logistic regression analysis (both unadjusted and adjusted) to investigate the predictors in the addiction and non-addiction groups while controlling the effects of age (1 = the addiction group, 0 = the non-addiction group). We found significant relationships between IA and negative coping styles (*P* < 0.05), acceptance of self and others (*P* = 0.001), and state anxiety levels (*P* = 0.003) (Table 3) in adjusted results indicating that these three factors had a significant association with more severe IA. In addition, we also conducted the regression analysis using a backward method and obtained results (Supplementary Table 5).

**Table 3:** Factors related to Internet addiction (*n* = 1,049).

**Regression factors**	**B**		**Wald test**		***P***		**Exp (B)**		**95%CI**	
	**Adjusted**	**Unadjusted**	**Adjusted**	**Unadjusted**	**Adjusted**	**Unadjusted**	**Adjusted**	**Unadjusted**	**Adjusted**	**Unadjusted**
State anxiety	0.032	0.055	8.655	52.191	0.003	<0.001	1.032	1.056	1.011–1.054	1.041–1.072
Trait anxiety	0.004	0.053	0.089	40.105	0.766	<0.001	1.004	1.054	0.980–1.028	1.037–1.071
Acceptance of self and others	−0.029	−0.047	10.561	36.970	0.001	<0.001	0.972	0.954	0.955–0.989	0.939–0.968
Acceptance by others	−0.003	−0.086	0.013	16.311	0.909	<0.001	0.997	0.917	0.948–1.048	0.879–0.956
Positive coping style	−0.230	−0.555	1.967	14.883	0.161	<0.001	0.794	0.574	0.576–1.096	0.433–0.761
Negative coping style	0.750	0.953	26.615	48.245	<0.001	<0.001	2.116	2.594	1.592–2.814	1.982–3.395
Gender	0.217	0.164	2.305	1.493	0.129	0.222	1.242	1.178	0.939–1.643	0.906–1.533

## Discussion

Our study focuses on exploring the relationship between IA and gender, coping styles, anxiety, and acceptance in the first-year college students in the South China. The results disclosed that males had higher scores in CIAS than females indicating that male might be a risk factor for IA, which is consistent with previous findings (12, 21). Among the participants, male first-year college students experienced less acceptance of self and others and acceptance by others and adopted less negative coping styles than their female counterparts.

Some possibilities might explain these differences. First, we found that males had a higher risk of developing IA than females. Previous studies revealed that males are more likely keen on the Internet for games, cybersex, and gambling to pursue feelings of achievement than females (48, 49). In addition to academic purposes, males tend to use the Internet for online games and information searching (50). By contrast, females tend to use the Internet for writing blogs, chatting, updating personal homepage, sending messages, and searching for information (51). Tateno et al. demonstrated that more males use the Internet for games, whereas more females use the Internet for social networking services, including Facebook and Twitter (52). Moon et al. revealed that males are more likely than females to engage in drug use to improve social bonding and self-esteem (53). Although previous studies produced inconsistent results regarding the gender effects on IA, most studies in China reported that IA is significantly higher in males than in females which is consistent with our result (12). Shek and Yu revealed that gender difference remains a factor for a high Internet addictive behavior in high school teenagers in Hong Kong (21). IA studies also showed that males have a general loss of control over their ability to limit their usage and engaged themselves in online gaming and Internet relay chats (50, 54, 55). Such activities could increase self-image by providing opportunities to interact with people with similar interests and pursue strong and intelligent self-identification, both of which augment the additive risk for males (39). Males also tend to be unwilling to communicate with others and seek help, resulting in a low exploitation rate of social support (56). Second, female college freshmen tend to have more family supervision than their male counterparts, which can limit the time for females spending online. Females are more likely to treasure off-line interpersonal relationships and are usually more cautious to new relationships online (57, 58). Some females may also make boyfriends online. Nevertheless, it may not conduce to IA because they would like to spend more time nose to nose (51). Females are often taught to build a considerable, passive, and graceful self-image since childhood (55, 59). Thus, they often represent acceptance of self and others and acceptance by others and practice negative coping styles in daily life.

Regarding psychological characteristics, the correlation analysis and logistic regression results in the present study showed that IA has a significant association with negative coping style, acceptance of self and others, and anxiety levels. This finding is consistent with previous studies that IA has a significant relation to emotional and behavior disorders, including anxiety, depression, obsessive-compulsive, aggressive, and avoidance behaviors (7, 27). The symptoms of pathological Internet use and emotional troubles could lead to a vicious cycle that favors the continuation of pathological Internet use (28). Ko et al. identified social phobia, depression, and emotional troubles as predictors of IA (60). Kitazawa et al. also revealed that anxiety and depression might predict IA in young adults (42). A longitudinal study revealed a bidirectional relationship between psychiatric symptoms and IA in college students after one-year follow up (37). The addiction group exhibited more negative emotions, including state and trait anxiety levels compared with the non-addiction group in the present study, indicating that those individuals with IA have a high risk of anxious in daily life, which is consistent with previous studies (24, 25, 27). Individuals with long-term IA have a low level of mental health, given that their condition leads to a lack of interpersonal interaction, which is a dangerous element for mental illness (56). As for anxiety, more Internet use has an association with an individual's shrinking social circle, low self-esteem, loneliness, and low life satisfaction (55). As the level of social anxiety increases, adults will spend less time in their interpersonal relationships and invest more time in the Internet, implying a great risk of developing IA (54).

The addiction group experienced little acceptance of self and others and acceptance by others. These individuals adopt negative coping styles rather than positive coping styles when they encounter some obstacles. Wei-Po showed that using less effective stress coping styles might induce high risk of IA, significant depression, and suicide attempts in college students after 1 year follow up (30). Individuals with negative coping styles, such as avoidance coping style, have a positive association with IA (29). Tonioni et al. and Chou et al. have reported relationship between inappropriate coping strategies and IA (61, 62). Less effective coping styles might lead to difficulties in the real world, and worsen the emotional state in young people (30). The risk of suicide might augment in a vicious cycle of ineffective coping styles and negative emotions (30). Of note, correlation coefficients were very weak (rs < 0.20) although the *p*-values were significant for correlation analysis because of the high number of participants in our study. Usually correlation coefficients reflect that *r* < 0.20 is very weak, and 0.20–0.40 is weak correlation. Our results show significant relationships between IA and negative coping style, acceptance of self and others, and state anxiety by using a logistic regression. Thus, health practitioners and scholars should pay attention to these three factors when consulting with Internet addicts or doing some researches on IA.

The Internet provides many benefits to its users, which tempts an increasing number of college students to become immersed in it. Previous studies have revealed that parental guidance of Internet behavior is associated with IA, especially when parents rarely communicate with regards to Internet use, lack of rules regarding Internet time (63) and lack of rules regarding Internet use (64). Other family variables, including parental relationship (65), perceived parental monitoring (66), and family conflict (66, 67), were also related to IA. Our results have the following suggestions to address such a problem. First, parents should guide their children to ideal Internet use and effectively monitor their children's Internet use based on their educational knowledge of the Internet (51). Regardless of people's perspectives of it, the Internet has entered most people's lives and becomes an important part of their lifestyles. Parents should encourage their children to use the Internet reasonably and avoid the negative points of the Internet if possible. Second, young children should form a good habit of using the Internet “positively” under the supervision of their parents. Parents were required to establish an Internet use plan for their children to let them know the severity of IA (68). Third, parents should also communicate with their children to understand their needs and invest more time to help relieve their inner troubles (69). Finally, Internet addicts should consult with psychologists and psychiatrists and jump the traces of the Internet as early as possible.

Several limitations in the present study should be considered. First, the cross-sectional design in the present study could not allow inferring cause–effect relationship between IA and its related factors. Second, we collected data from a single college, and the findings might not generalize to all China or all university students. Third, this study did not explore the details of the participants' Internet use. We were unclear which games, social networking services, or other scanning habits might significantly change Internet habits or dependence in the freshmen. Fourth, although the CIAS involves an assessment of time management to the Internet, we did not measure how much time the subjects spent in the Internet, and the usage of media device, such as game, smartphone, and laptop was not recorded in the present study. Fifth, the assessments are self-rating scales, which could easily generate potential information deviation. Sixth, previous studies have revealed that the acceptable alpha values range from 0.70 to 0.95 (59, 60). The Cronbach's alphas for SCSQ have a low internal consistency reliability in the present study (<0.7), suggesting that some items may need to be revised or deleted. Hence, the result should be interpreted with caution.

## Conclusion

The present study revealed that IA is more common in male first-year college students than in their female counterparts, and males have a greater risk of developing IA than females. Although some studies have shown opposite results or no difference, research evidence (12, 21) has revealed that men tend to have a higher risk of IA. Our results are in line with the earlier and more recent findings that IA is high in men. State and trait anxiety levels, negative coping styles, little acceptance by others, little acceptance of self and others, and non-adoption of positive coping styles have an association with more severe IA in college freshmen. Thus, scholars and health practitioners should consider gender differences when formulating strategies to prevent IA, so that it could offer significative guidance for clinicians, parents and educators. Except for parental intervention and supervision, schools should encourage students to take part in more social practice activities and instruct students to use the Internet with a good habit. We should focus on the social education of IA in future research. In addition, future studies with more objective methods and lager sample are required to evaluate IA and related risk factors in high-risk populations.

## Data Availability Statement

All datasets presented in this study are included in the article/Supplementary Material.

## Ethics Statement

The study involving human participants was reviewed and approved by the Ethics Committee of the Second Xiangya Hospital of Central South University, and was also approved by the university's institutional review board and executed in line with the Helsinki Declaration. All the participants provided informed consent. An additional informed consent was obtained from the parents or guardians for those aged below 18 years old.

## Author Contributions

XS is mainly responsible for essay writing and conducting the study. YO, YD, and HY managed and analyzed the data. WG, JC, and JZ designed the study. All authors contributed to the article and approved the submitted version.

## Conflict of Interest

The authors declare that the research was conducted in the absence of any commercial or financial relationships that could be construed as a potential conflict of interest.

## References

[1] Greydanus DE Greydanus MM. Internet use, misuse, and addiction in adolescents: current issues and challenges. Int J Adolesc Med Health. (2012) 24:283–9. 10.1515/ijamh.2012.041 23183727

[2] WHO. Public Health Implications of Excessive Use of the Internet, Computers, Smartphones and Similar Electronic Devices: Meeting Report, Main Meeting Hall, Foundation for Promotion of Cancer Research, National Cancer Research Centre, Tokyo, Japan. Geneva: World Health Organization (2015). p. 27–9.

[3] Ofole NM Babatunde OO. Internet addiction among undergraduates in university of Ibadan: imperative for counselling intervention. Afr J Psychol Study Soc Issues. (2015) 18:3.

[4] Li W O'Brien JE Snyder SM Howard MO. Diagnostic criteria for problematic internet use among U.S. University students: a mixed-methods evaluation. PLoS ONE. (2016) 11:e0145981. 10.1371/journal.pone.0145981 26751569 PMC4709169

[5] Chou C Hsiao MC. Internet addiction, usage, gratification, and pleasure experience: the Taiwan college students' case. Comp Educ. (2000) 35:65–80. 10.1016/S0360-1315(00)00019-1

[6] Kubey RW Lavin MJ Barrows JR. Internet use and collegiate academic performance decrements: early findings. J Commun. (2001) 51:366e82. 10.1111/j.1460-2466.2001.tb02885.x

[7] Park S Hong KE Park EJ Ha KS Yoo HJ. The association between problematic internet use and depression, suicidal ideation and bipolar disorder symptoms in Korean adolescents. Aust N ZJ Psychiatry. (2013) 47:153–9. 10.1177/0004867412463613 23047959

[8] Ko CH Yen JY Yen CF Chen CS Chen CC. The association between internet addiction and psychiatric disorder: a review of the literature. Eur Psychiatry. (2012) 27:1–8. 10.1016/j.eurpsy.2010.04.011 22153731

[9] Bernardi S Pallanti S. Internet addiction: a descriptive clinical study focusing on comorbidities and dissociative symptoms. Compr Psychiatry. (2009) 50:510–6. 10.1016/j.comppsych.2008.11.011 19840588

[10] Ko CH Yen JY Liu SC Huang CF Yen CF. The associations between aggressive behaviors and internet addiction and online activities in adolescents. J Adolesc Health. (2009) 44:598–605. 10.1016/j.jadohealth.2008.11.011 19465325

[11] Ekenze SO Okafor CI Ekenze OS Nwosu JN Ezepue UF. The value of internet tools in undergraduate surgical education: perspective of medical students in a developing country. World J Surg. (2017) 41:672–80. 10.1007/s00268-016-3781-x 27812808

[12] Shao YJ Zheng T Wang YQ Liu L Chen Y Yao YS. Internet addiction detection rate among college students in the People's Republic of China: a meta-analysis. Child Adolesc Psychiatry Ment Health. (2018) 12:25. 10.1186/s13034-018-0231-6 29849754 PMC5970523

[13] Chen YL Chen SH Gau SS. ADHD and autistic traits, family function, parenting style, and social adjustment for internet addiction among children and adolescents in Taiwan: a longitudinal study. Res Dev Disabil. (2015) 39:20–31. 10.1016/j.ridd.2014.12.025 25617844

[14] Nakayama H Higuchi S. Internet addiction. Nihon Rinsho. (2015) 73:1559–66. 26394521

[15] Li R Shi G Ji J Wang H Wei W Meng W. A 2-year longitudinal psychological intervention study on the prevention of internet addiction in junior high school students of Jinan city. Biomed Res. (2017) 28:10033–8.

[16] Kuss DJ Griffiths MD Karila L Billieux J. Internet addiction: a systematic review of epidemiological research for the last decade. Curr Pharm Des. (2014) 20:4026–52. 10.2174/13816128113199990617 24001297

[17] Sayili U Vehid S Erginöz E. Problematic internet use in turkish high school students: prevalence and related factors. Am J Health Behav. (2021) 45:31–43. 10.5993/AJHB.45.1.3 33402236

[18] Shen Y Wang L Huang C Guo J De Leon SA Lu J. Sex differences in prevalence, risk factors and clinical correlates of internet addiction among Chinese college students. J Affect Disord. (2020) 279:680–6. 10.1016/j.jad.2020.10.054 33190119

[19] Alamgir KM Faizania S Ahmed RT. Effect of gender and physical activity on internet addiction in medical students. Pak J Med Sci. (2017) 33:191–4. 10.12669/pjms.331.11222 28367198 PMC5368307

[20] Marzill E Cerniglia L Ballarotto G Cimino S. Internet addiction among young adult university students: the complex interplay between family functioning, impulsivity, depression, and anxiety. Int J Environ Res Public Health. (2020) 17:8231. 10.3390/ijerph17218231 33171742 PMC7664422

[21] Shek DTL Yu L. Adolescent internet addiction in hong kong: prevalence, change, and correlates. J Pediatr Adolesc Gynecol. (2016) 29:S22–30. 10.1016/j.jpag.2015.10.005 26461526

[22] Chiu S Hong FY Chiu SL. An analysis on the correlation and gender difference between college students' internet addiction and mobile phone addiction in Taiwan. Isrn Addict. (2013) 2013:360607. 10.1155/2013/360607 25938115 PMC4392944

[23] Yan C Kang Y Gong W He L Jin Y Zhu X. Investigation on internet addiction disorder in adolescents in Anhui, People's Republic of China. Neuropsychiatr Dis Treat. (2016) 12:2233–36. 10.2147/NDT.S110156 27621633 PMC5010169

[24] Floros G Siomos K Stogiannidou A Giouzepas I Garyfallos G. The relationship between personality, defense styles, internet addiction disorder, and psychopathology in college students. Cyberpsychol Behav Soc Netw. (2014) 17:672–6. 10.1089/cyber.2014.0182 25225916

[25] Ho RC Zhang MW Tsang TY Toh AH Pan F Lu Y. The association between internet addiction and psychiatric co-morbidity: a meta-analysis. BMC Psychiatry. (2014) 14:183. 10.1186/1471-244X-14-183 24947851 PMC4082374

[26] Davis C Loxton NJ. Addictive behaviors and addiction-prone personality traits: associations with a dopamine multilocus genetic profile. Addict Behav. (2013) 38:2306–12. 10.1016/j.addbeh.2013.02.012 23584190

[27] Jang KS Hwang SY Choi JY. Internet addiction and psychiatric symptoms among Korean adolescents. J School Health. (2010) 78:165–71. 10.1111/j.1746-1561.2007.00279.x 18307612

[28] Strittmatter E Parzer P Brunner R Fischer G Durkee T Carli V. A 2-year longitudinal study of prospective predictors of pathological internet use in adolescents. Eur Child Adolesc Psychiatry. (2016) 25:725–34. 10.1007/s00787-015-0779-0 26526444

[29] Mcnicol ML Thorsteinsson EB. Internet addiction, psychological distress, and coping responses among adolescents and adults. Cyberpsychol Behav Soc Netw. (2017) 20:296–304. 10.1089/cyber.2016.0669 28414517 PMC5485234

[30] Wei-Po C Cheng-Fang Y Liu TL. Predicting effects of psychological inflexibility/experiential avoidance and stress coping strategies for internet addiction, significant depression, and suicidality in college students: a prospective study. Int J Environ Res Public Health. (2018) 15:788. 10.3390/ijerph15040788 29670025 PMC5923830

[31] Soule LC Shell LW Kleen BA. Exploring internet addiction: demographic characteristics and stereotypes of heavy internet users. Data Process Better Business Educ. (2003) 44:64–3. 10.1080/08874417.2003.11647553

[32] Young KS Rogers RC. The relationship between depression and internet addiction. Cyberpsychol Behav. (1998) 1:178–83. 10.1089/cpb.1998.1.25

[33] Young KS. Internet addiction a new clinical phenomenon and its consequences. Am Behav Sci. (2004) 48:402–15. 10.1177/0002764204270278

[34] Chen SH Weng LC Su YJ Wu H Yang PF. Development of Chinese Internet Addiction Scale and its psychometric study. Chinese J Psychol. (2003) 45:279–94. 10.1037/t44491-000

[35] Nie J Zhang W Chen J Li W. Impaired inhibition and working memory in response to internet-related words among adolescents with internet addiction: a comparison with attention-deficit/hyperactivity disorder. Psychiatry Res. (2016)236:28–34. 10.1016/j.psychres.2016.01.004 26778632

[36] Ko CH Liu TL Wang PW Chen CS Yen CF Yen JY. The exacerbation of depression, hostility, and social anxiety in the course of internet addiction among adolescents: a prospective study. Compr Psychiatry. (2014) 55:1377–84. 10.1016/j.comppsych.2014.05.003 24939704

[37] Lin YJ Hsiao RC Liu TL Yen CF. Bidirectional relationships of psychiatric symptoms with internet addiction in college students: a prospective study. J Formosan Med Assoc. (2020) 119:1093–100. 10.1016/j.jfma.2019.10.006 31653577

[38] Lau JT Gross DL Wu AM Cheng KM Lau MM. Incidence and predictive factors of internet addiction among Chinese secondary school students in Hong Kong: a longitudinal study. Soc Psychiatry Psychiatr Epidemiol. (2017) 52:657–67. 10.1007/s00127-017-1356-2 28417158

[39] Ko CH Yen CF Yen JY Chen CC Yen CN Chen SH. Screening for internet addiction: an empirical study on cut-off points for the Chen internet addiction scale. Kaohsiung J Med Sci. (2005) 21:545–51. 10.1016/S1607-551X(09)70206-2 16670046 PMC11918109

[40] Chang FC Chiu CH Lee CM Chen PH Miao NF. Predictors of the initiation and persistence of internet addiction among adolescents in Taiwan. Addict Behav. (2014) 39:1434–40. 10.1016/j.addbeh.2014.05.010 24930050

[41] Mizuguchi T Shimonaka J Nakazato K. Japanese version STAI. Kyoto: Sankyoubou (1991). pp. 1–6. (in Japanese).

[42] Kitazawa M Yoshimura M Murata M Sato-Fujimoto Y Hitokoto H Mimura M. Associations between problematic Internet use and psychiatric symptoms among university students in Japan. Psychiatry Clin Neurosci. (2018) 72:531–9. 10.1111/pcn.12662 29652105

[43] Fey WF. Acceptance by others and its relation to acceptance of self and others: a reevaluation. J Abn Soc Pocial Psychol. (1955) 50:274–6. 10.1037/h0046876 14366895

[44] Minn EJ. A Study of Acceptance by Others and Its Relation to Acceptance of Self and Others Among Selected Student Nurses. Doctoral dissertation. McNeese State University (2020).

[45] Xie YN. Development of simplified coping style questionnaire. Chin J Clin Psychol. (1998) 6:114–5.

[46] Yi J Zhong B Yao S. Health-related quality of life and influencing factors among rural left-behind wives in Liuyang, China. BMC Womens Health. (2014) 14:67. 10.1186/1472-6874-14-67 24886024 PMC4024212

[47] Kraaij V Garnefski N Maes S. The joint effects of stress, coping, and coping resources on depressive symptoms in the elderly. Anxiety Stress Coping. (2002) 15:163–77. 10.1080/10615800290028468

[48] Fattore L Melis M Fadda P Fratta W. Sex differences in addictive disorders. Front Neuroendocrinol. (2014) 35:272–84. 10.1016/j.yfrne.2014.04.003 24769267

[49] Johansson A Gotestam KG. Internet addiction: characteristics of a questionnaire and prevalence in Norwegian youth (12–18 years). Scand J Psychol. (2004) 45:223–9. 10.1111/j.1467-9450.2004.00398.x 15182240

[50] Joiner R Gavin J Brosnan M Cromby J Gregory H Guiller J. Gender, internet experience, internet identification, and internet anxiety: a ten-year followup. Cyberpsychol Behav Soc Netw. (2012) 15:370–2. 10.1089/cyber.2012.0033 22690795

[51] Heo J Oh J Subramanian SV Kim Y Kawachi I. Addictive internet use among korean adolescents: a national survey. PLoS ONE. (2014) 9:e87819. 10.1371/journal.pone.0087819 24505318 PMC3914839

[52] Tateno M Teo AR Shirasaka T Tayama M Watabe M Kato TA. Internet addiction and self-evaluated attention-deficit hyperactivity disorder traits among Japanese college students. Psychiatry Clin Neurosci. (2016) 70:567–72. 10.1111/pcn.12454 27573254 PMC5573248

[53] Moon DG Hecht ML Jackson KM Spellers RE. Ethnic and gender differences and similarities in adolescent drug use and refusals of drug offers. Subst Use Misuse. (1999) 34:1059–83. 10.3109/10826089909039397 10359222

[54] Baloglu M Ozteke KH Kesici S. Gender differences in and the relationships between social anxiety and problematic internet use: canonical analysis. J Med Int Res. (2018) 20:e33. 10.2196/jmir.8947 29367182 PMC5803528

[55] Ko CH Yen JY Chen CC Chen SH Yen CF. Gender differences and related factors affecting online gaming addiction among Taiwanese adolescents. J Nerv Ment Dis. (2005) 193:273–7. 10.1097/01.nmd.0000158373.85150.57 15805824

[56] Zhou XY Xu F Wei XL Zhao L Pang BH Chang JB. Internet addiction disorder and mental health in the university students in Yan'an City. China J Health Psychol. (2015) 23:1506–9. 10.13342/j.cnki.cjhp.2015.10.019

[57] Kim H Kim E Min K Shin J Lee S. International conference on socialization in adolescence III on the relationship of parents-children, teachers students, and among peers. In: National Youth Policy Institute, editor. International Conference on Socialization in Adolescence. (2007).

[58] Gross EF. Adolescent internet use: what we expect, what teens report. J Appl Dev Psychol. (2004) 25:633–49. 10.1016/j.appdev.2004.09.005

[59] McKenna KY Green AS Gleason ME. Relationship formation on the internet: what's the big attraction? J Soc Issues. (2002) 58:9–31. 10.1111/1540-4560.00246

[60] Ko CH Yen JY Chen CS Yeh YC Yen CF. Predictive values of psychiatric symptoms for internet addiction in adolescents: a 2-year prospective study. Arch Pediatr Adolesc Med. (2009) 163:937–43. 10.1001/archpediatrics.2009.159 19805713

[61] Tonioni F Mazza M Autullo G Cappelluti R Catalano V Marano G. IsInternetaddictionapsychopathologicalconditiondistinctfrompathological gambling? Addict Behav. (2014) 39:1052–6. 10.1016/j.addbeh.2014.02.016 24630825

[62] Chou WP Ko CH Kaufman EA Crowell SE Hsiao RC Wang PW. Association of stress coping strategies with internet addiction in college students: the moderating effect of depression. Compr Psychiatry. (2015) 62:27–33. 10.1016/j.comppsych.2015.06.004 26343464

[63] van den Eijnden RJ Spijkerman R Vermulst AA van Rooij TJ Engels RC. Compulsive internet use among adolescents: bidirectional parent-child relationships. J Abnorm Child Psychol. (2010) 38:77–89. 10.1007/s10802-009-9347-8 19728076 PMC2809946

[64] Mythily S Qiu S Winslow M. Prevalence and correlates of excessive internet use among youth in Singapore. Ann Acad Med Singap. (2008) 37:9–14. 18265891

[65] Siomos K Floros G Fisoun V Evaggelia D Farkonas N Sergentani E. Evolution of internet addiction in Greek adolescent students over a two-year period: the impact of parental bonding. Eur Child Adolesc Psychiatry. (2012) 21:211–9. 10.1007/s00787-012-0254-0 22311146

[66] Yen CF Ko CH Yen JY Chang YP Cheng CP. Multi-dimensional discriminative factors for internet addiction among adolescents regarding gender and age. Psychiatry Clin Neurosci. (2009) 63:357–64. 10.1111/j.1440-1819.2009.01969.x 19566768

[67] Wang H Zhou X Lu C Wu J Deng X Hong L. Problematic internet use in high school students in Guangdong Province, China. PLoS ONE. (2011) 6:e19660. 10.1371/journal.pone.0019660 21573073 PMC3089638

[68] Lam LT. Parental mental health and internet addiction in adolescents. Addict Behav. (2015) 42:20–3. 10.1016/j.addbeh.2014.10.033 25462649

[69] Lin YH Gau SS. Association between morningness- eveningness and the severity of compulsive internet use: the moderating role of gender and parenting style. Sleep Med. (2013) 14:1398–404. 10.1016/j.sleep.2013.06.015 24157101

