# Supplementary material for: Associations Between Internet Addiction and Gender, Anxiety, Coping Styles and Acceptance in University Freshmen in South China
Source: Front Psychiatry. 2021 May 31;12:558080. doi: 10.3389/fpsyt.2021.558080 (PMC8200474; doi:10.3389/fpsyt.2021.558080)
Supplement: Supplementary file 1 [file Data_Sheet_1.docx]

Table S1 Demographic characteristics of the subjects

|  | Males | Females | P |
| --- | --- | --- | --- |
| [Single-child](javascript:;) [family](javascript:;)(Yes/No) | 40.76%/59.24%(785/1141) | 55.53%/44.47(753/603) | <0.001^a^ |
| Age | 19.91±0.86 | 19.73±0.73 | <0.001^b^ |
| Adoptive parents(Yes/No) | 2.67%/ 97.33% (53/ 1934) | 3.56%/97.44%(49/ 1328) | 0.138^a^ |
| Father alive (Yes/No) | 98.23%/1.77% (1888/34) | 98.44%/1.56%(1328/21) | 0.642^a^ |
| Mother alive (Yes/No) | 99.22%/0.78 (1916/15) | 99.48%/0.52%(1349/7) | 0.367^a^ |

^a^P values were obtained by chi-square tests.

^b^P values were obtained by two-sample t-tests.

CIAS = Chinese Internet Addiction Scale.

Table S2 Psychological characteristics of college freshmen in male and female groups

|  | Male | Female | P |
| --- | --- | --- | --- |
| CIAS | 39.91±11.67 | 37.23±10.61 | <0.001 |
| State anxiety | 36.75±8.68 | 36.29±8.58 | 0.135 |
| Trait anxiety | 40.50±7.65 | 40.00±7.50 | 0.061 |
| Acceptance of self and others | 59.79±8.62 | 61.52±8.27 | <0.001 |
| Acceptance by others | 17.42±2.90 | 17.74±3.11 | 0.003 |
| Positive coping style | 1.96±0.42 | 1.97±0.43 | 0.066 |
| Negative coping style | 0.94±0.45 | 0.98±0.47 | 0.004 |

P values were obtained by two-sample t-tests.

CIAS = Chinese Internet Addiction Scale.

Table S3 Psychological characteristics of college freshmen in male and female groups using Wilcoxon tests

|  | Male | Female | P | Z |
| --- | --- | --- | --- | --- |
| CIAS | 39.91±11.67 | 37.23±10.61 | <0.001 | -6.184 |
| State anxiety | 36.75±8.68 | 36.29±8.58 | 0.115 | -1.575 |
| Trait anxiety | 40.50±7.65 | 40.00±7.50 | 0.069 | -1.821 |
| Acceptance of self and others | 59.79±8.62 | 61.52±8.27 | <0.001 | -5.890 |
| Acceptance by others | 17.42±2.90 | 17.74±3.11 | 0.004 | -2.859 |
| Positive coping style | 1.96±0.42 | 1.97±0.43 | 0.140 | -1.477 |
| Negative coping style | 0.94±0.45 | 0.98±0.47 | 0.003 | -2.925 |

P values were obtained by Wilcoxon tests of two independent samples.

CIAS = Chinese Internet Addiction Scale.

Table S4 Simple correlation coefficients across all variables

|  |  | Sex | [Single-child](javascript:;) [family](javascript:;) | Adoptive parents | Father alive | Mother alive | Positive coping style | Negative coping style | Acceptance of self and others | Acceptance by others | State anxiety | Trait anxiety | CIAS score |
| --- | --- | --- | --- | --- | --- | --- | --- | --- | --- | --- | --- | --- | --- |
| Sex | R | - | -0.145 | 0.025 | -0.008 | -0.016 | 0.029 | 0.047 | 0.097 | 0.051 | -0.025 | -0.032 | -0.116 |
|  | P | - | ＜0.001 | 0.141 | 0.639 | 0.366 | 0.089 | 0.007 | ＜0.001 | 0.003 | 0.147 | 0.061 | ＜0.001 |
| [Single-child](javascript:;)  [family](javascript:;) | R | -0.145 | - | 0.044 | 0.021 | 0.017 | -0.022 | 0.001 | -0.068 | -0.083 | 0.080 | 0.081 | -0.021 |
|  | P | ＜0.001 | - | 0.011 | 0.241 | 0.335 | 0.207 | 0.964 | ＜0.001 | ＜0.001 | ＜0.001 | ＜0.001 | 0.226 |
| Adoptive  parents | R | 0.025 | 0.044 | - | 0.107 | 0.235 | 0.019 | -0.003 | 0.008 | 0.017 | 0.003 | 0.002 | -0.013 |
|  | P | 0.141 | 0.011 | - | ＜0.001 | ＜0.001 | 0.269 | 0.881 | 0.639 | 0.320 | 0.874 | 0.910 | 0.460 |
| Father alive | R | -0.008 | 0.021 | 0.107 | - | 0.144 | 0.012 | 0.009 | -0.002 | -0.016 | 0.013 | -0.006 | 0.016 |
|  | P | 0.639 | 0.241 | ＜0.001 | - | ＜0.001 | 0.478 | 0.613 | 0.891 | 0.348 | 0.463 | 0.747 | 0.356 |
| Mother alive | R | -0.016 | 0.017 | 0.235 | 0.144 | - | 0.027 | -0.027 | 0.011 | -0.025 | 0.008 | 0.006 | 0.022 |
|  | P | 0.366 | 0.335 | ＜0.001 | ＜0.001 | - | 0.116 | 0.118 | 0.513 | 0.150 | 0.655 | 0.737 | 0.198 |
| Positive coping  style | R | 0.029 | -0.022 | 0.019 | 0.012 | 0.027 | - | -0.003 | 0.126 | 0.158 | -0.292 | -0.335 | -0.075 |
|  | P | 0.089 | 0.207 | 0.269 | 0.478 | 0.116 | - | 0.857 | ＜0.001 | ＜0.001 | ＜0.001 | ＜0.001 | ＜0.001 |
| Negative coping style | R | 0.047 | 0.001 | -0.003 | 0.009 | -0.027 | -0.003 | - | -0.214 | -0.155 | 0.225 | 0.273 | 0.159 |
|  | P | 0.007 | 0.964 | 0.881 | 0.613 | 0.118 | 0.857 | - | ＜0.001 | ＜0.001 | ＜0.001 | ＜0.001 | ＜0.001 |
| Acceptance of  self and others | R | 0.097 | -0.068 | 0.008 | -0.002 | 0.011 | 0.126 | -0.214 | - | 0.439 | -0.296 | -0.341 | -0.152 |
|  | P | ＜0.001 | ＜0.001 | 0.639 | 0.891 | 0.513 | ＜0.001 | ＜0.001 | - | ＜0.001 | ＜0.001 | ＜0.001 | ＜0.001 |
| Acceptance by  others | R | 0.051 | -0.083 | 0.017 | -0.016 | -0.025 | 0.158 | -0.155 | 0.439 | - | -0.316 | -0.353 | 0.099 |
|  | P | 0.003 | ＜0.001 | 0.320 | 0.348 | 0.150 | ＜0.001 | ＜0.001 | ＜0.001 | - | ＜0.001 | ＜0.001 | ＜0.001 |
| State anxiety | R | -0.025 | 0.080 | 0.003 | 0.013 | 0.008 | -0.292 | 0.225 | -0.296 | -0.316 | - | 0.710 | 0.166 |
|  | P | 0.147 | ＜0.001 | 0.874 | 0.463 | 0.655 | ＜0.001 | ＜0.001 | ＜0.001 | ＜0.001 | - | ＜0.001 | ＜0.001 |
| Trait anxiety | R | -0.032 | 0.081 | 0.002 | -0.006 | 0.006 | -0.335 | 0.273 | -0.341 | -0.353 | 0.710 | - | 0.164 |
|  | P | 0.061 | ＜0.001 | 0.910 | 0.747 | 0.737 | ＜0.001 | ＜0.001 | ＜0.001 | ＜0.001 | ＜0.001 | - | ＜0.001 |

CIAS = Chinese Internet Addiction Scale.

Table S5 Backward LR method Logistic Regression Model

| Regression factors | B | Wald test | P | Exp(B) | 95%CI for Exp(B) | |
| --- | --- | --- | --- | --- | --- | --- |
|  |  |  |  |  | Lower | Upper |
| State anxiety | 0.038 | 21.642 | <0.001 | 1.038 | 1.022 | 1.055 |
| Acceptance of self and others | -0.031 | 13.604 | <0.001 | 0.970 | 0.954 | 0.986 |
| Negative coping style | 0.722 | 25.833 | <0.001 | 2.059 | 1.558 | 2.720 |
